# Innate immunity in pancreatic cancer: Lineage tracing and function

**DOI:** 10.3389/fimmu.2022.1081919

**Published:** 2023-01-16

**Authors:** Longyun Ye, Saimeng Shi, Wei Chen

**Affiliations:** ^1^ Department of Pancreatic Surgery, Fudan University Shanghai Cancer Center, Shanghai, China; ^2^ Department of Oncology, Shanghai Medical College, Fudan University, Shanghai, China; ^3^ Shanghai Pancreatic Cancer Institute, Shanghai, China; ^4^ Pancreatic Cancer Institute, Fudan University, Shanghai, China; ^5^ Institute of Clinical Medicine Research, Zhejiang Provincial People's Hospital, Hangzhou Medical College, Hangzhou, China; ^6^ Cancer Institute of Integrated Traditional Chinese and Western Medicine, Zhejiang Academy of Traditional Chinese Medicine, Tongde Hospital of Zhejiang Province, Hangzhou, China

**Keywords:** pancreatic cancer, tumor immune microenvironment, innate immune lymphocyte, lineage tracing, innate immunity

## Abstract

Increasingly, patients with gastrointestinal tumors can benefit from immunotherapy, but not patients with pancreatic cancer. While this lack of benefit has been attributed to lower T-cell infiltration in pancreatic cancer, other studies have demonstrated the presence of numerous T cells in pancreatic cancer, suggesting another mechanism for the poor efficacy of immunotherapy. Single-cell RNA sequencing studies on the pancreatic cancer immune microenvironment have demonstrated the predominance of innate immune cells (e.g., macrophages, dendritic cells, mast cells, and innate immune lymphoid cells). Therefore, in-depth research on the source and function of innate immune lymphocytes in pancreatic cancer could guide pancreatic cancer immunotherapy.

## 1 Introduction

**Figure 1:**
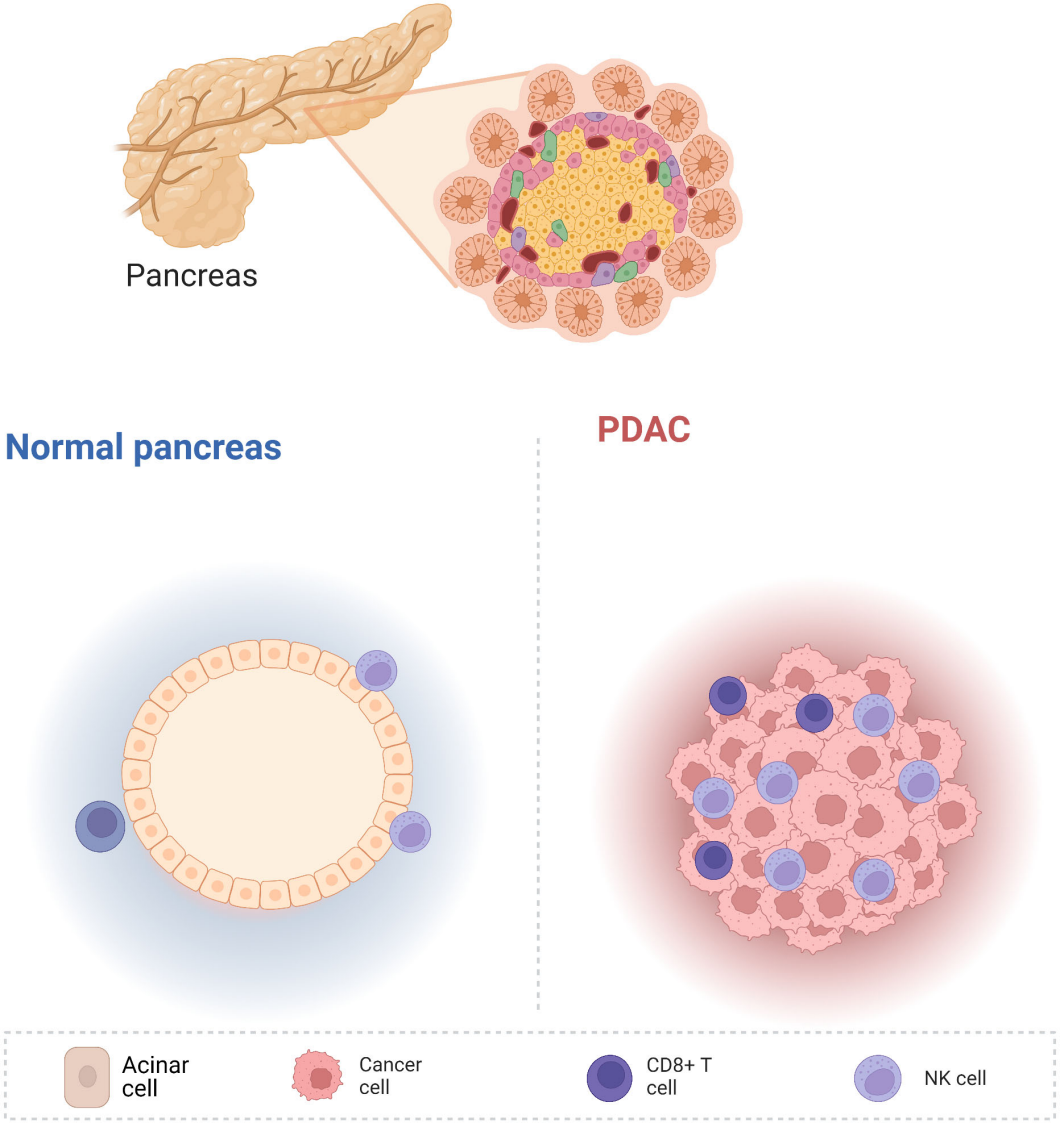
Compared with other digestive tract organs, the non-cancerous pancreas is relatively clean and contains very few immune cells. Nevertheless, pancreatic cancer development is accompanied by the infiltration of numerous immune cells, including innate and adaptive immune cells.

The pancreas is located in the retroperitoneum and connects to the digestive tract only *via* the pancreatic duct, without direct contact with the external environment. Therefore, the pancreas is relatively “clean” compared with the other digestive organs. Therefore, normal pancreatic tissue contains very few lymphocytes, which has been confirmed by both our research (1) and that of other researchers (2). However, pancreatic cancer initiation and development are followed by substantial immune cell infiltration (**Figure 1**). Fractionation of the immune cells in pancreatic adenocarcinoma (PDAC) revealed a greater abundance of innate immune cells than adaptive immune cells (2). Innate immune cells are the primary responders to inflammation; therefore, a large proportion of macrophages, myeloid-derived suppressor cells (MDSCs), and dendritic cells (DCs) are observed in pancreatic cancer. The tumor microenvironment includes excessive fibrosis, which further hampers the infiltration of adaptive immune cells.

Current immunotherapy, including immune checkpoint blockade, chimeric antigen receptor-T, and T-cell receptor-T, has shown a low response rate in PDAC so far. The non-responsiveness to immunotherapy in pancreatic cancers can be explained by these tumors being less immunogenic (harboring fewer somatic mutations) compared with breast and lung tumors, which tend to have better immunotherapy responsive rates.

Furthermore, compared with adaptive immunity, innate immune cells do not require antigen presentation, and can be rapidly and directly activated by a large number of cytokines. Therefore, in light of the predominance infiltration, decreased antigenicity and instant activation, innate immunity might play a more important role than adaptive immunity in the PDAC immune microenvironment.

In this review, we categorize the innate immune lymphocytes in pancreatic cancer and describe their origin and function. We believe that in-depth study of the innate immune function and characterizing the pancreatic cancer immune microenvironment is crucial to improve the efficacy of pancreatic cancer immunotherapy.

## 2 Macrophages

**Figure 2:**
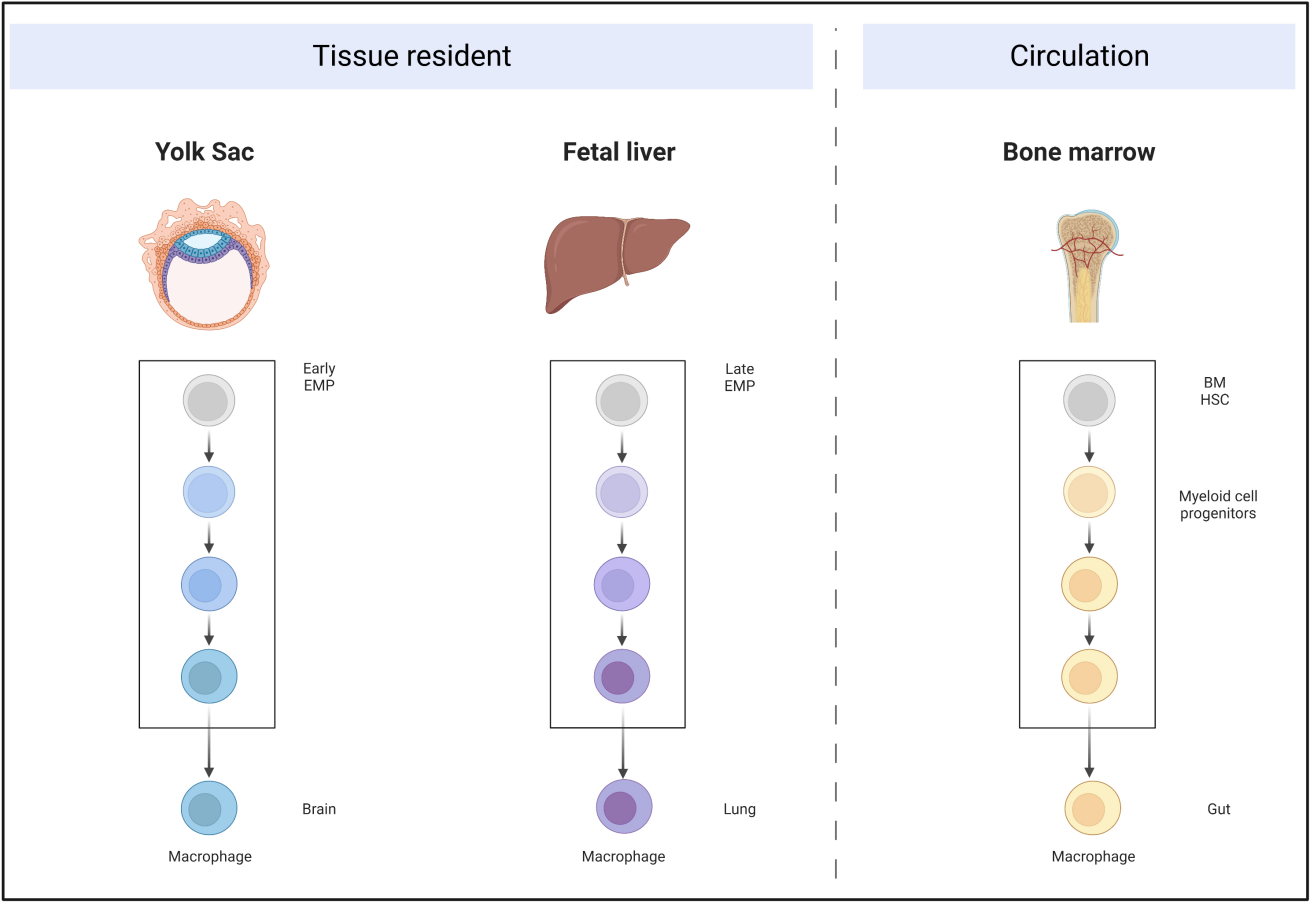
Self-proliferative tissue-resident macrophages originate from the yolk sac and fetal liver, migrate into the peripheral organs during embryonic development, and mature. Circulating macrophages are derived from bone marrow HSCs, which develop into myeloid-committed stem cells and further develop into macrophages.

Single-cell RNA sequencing (RNA-seq) data from Magliano’s group (2) demonstrated that macrophages are the dominant immune cells among the CD45^+^ population in PDAC. Previously, it was believed that peripheral tissue macrophages were derived from circulating monocytes, which originate from bone marrow progenitors. Recently, accumulating studies have demonstrated two independent origins for macrophages (3–7). The first comprises early erythroid-myeloid precursor cells (EMPs) of the yolk sac (embryonic day [E]7) or the late EMPs of the liver (E8–E9.5). The EMPs migrate into the peripheral tissues during the embryonic period, differentiate, and mature, and have self-renewal and maintenance functions in adults. The other macrophage origin is hematopoietic stem cells (HSCs) from the bone marrow that enter the peripheral tissues through the blood circulation, and then mature and differentiate into monocytes. The proportions of these two macrophage origins differ quite significantly between different organs (**Figure 2**).

In brain tissue, microglia are derived from the early EMPs of the yolk sac and are not replenished by circulating monocytes (3). By contrast, most adult intestinal macrophages are constantly replenished by peripheral monocytes, for which Bain et al. (7) demonstrated that the F4/80^hi^CD11b^lo^ subset (derived from embryonic precursors) was dominant in newborn mice, but was progressively lost in adult mice, in which almost all macrophages were F4/80^low^CD11b^hi^ (derived from the circulation).

Similarly, in the pancreas, macrophages have an obvious dual origin. Using lineage tracing mice (Flt3-Cre^YFP^; Flt3, Fms related receptor tyrosine kinase 3; YFP, yellow fluorescent protein) in which HSC-derived cells were YFP^+^ and embryo-derived cells were YFP^-^, Zhu et al. demonstrated that approximately 32% of macrophages in the pancreas were YFP^-^. Furthermore, they established orthotopic KPC (*LSL-Kras^G12D/+^LSL-Trp53^R172H/+^Pdx1-Cre*) tumor mice. Similar to the results in the non-tumorous pancreas, almost one-third of macrophages were YFP^-^, indicating that they were tissue-resident (8).

The cells from these two origins have entirely different development environments, which means that they might have different functions. Using *Ccr2* (encoding C-C motif chemokine receptor 2) and *Nur77* (encoding nuclear hormone receptor NUR/77) knockout mice, Zhu et al. demonstrated that bone marrow-derived macrophages were more prone to expressing human leucocyte antigen DR (HLA-DR), and its absence did not impair PDAC development (8). By contrast, embryonically derived tumor-associated macrophages (TAMs) exhibited unique pro-fibrotic activity and promoted cancer progression. Macrophages of these different origins appear to be distinguished by C-X-C motif chemokine receptor 4 (CXCR4) in human pancreatic cancer, where CXCR4-positive macrophages appear to be tissue-resident and have a pro-fibrotic effect. Bockorny et al. used the CXCR4 inhibitor, BL-8040, in combination with pembrolizumab and chemotherapy, which was initially successful; however, the specific mechanism is unknown (9). Whether *CXCR4* is merely a downstream marker or functions as a driving gene requires further exploration.

Traditionally, macrophage function is divided into the M1 and M2 types; however, this division might have some limitations. Single-cell RNA-seq data suggested that macrophage function appears to present as a continuous, rather than a separate state (10–12) and is highly susceptible to the influence of the microenvironment.

Macrophages are generally inclined to the M2 phenotype in pancreatic cancer. Their infiltration density was associated with disease progression, recurrence, and metastasis in patients with PDAC (13). TAM-derived interleukin (IL)6, tumor necrosis factor alpha (TNFα), and regulated upon activation, normally t-expressed, and presumably secreted (RANTES) could facilitate acinar-to-ductal metaplasia and pancreatic intraepithelial neoplasia development *via* Janus kinase (JAK)-signal transducer and activator of transcription3 (STAT3) (14) or nuclear factor kappa B (NF-κB) (15) signaling. Depletion of TAMs could dramatically decrease tumorigenesis (16). In addition, TAMs could also express a series of immunosuppressive factors (e.g., IL-10, transforming growth factor beta (TGF-β) and C-C motif chemokine ligand (CCL)2) and negative immune checkpoints (e.g., cytotoxic T-lymphocyte associated protein 4 (CTLA-4) and programmed cell death 1 ligand 1 (PD-L1)) to promote immune escape in PDAC (17). With regard to fibrosis, macrophages could secrete growth factors (e.g., TGFβ1), cytokines (e.g., TNFα) and chemokines (e.g., CCL2), which directly activate fibroblasts to form fibrous desmoplastic tissue (18). This interstitial fibrosis leads to extreme hypoxia, which in turn induces polarization toward the M2 phenotype, promotes the expression of vascular endothelial growth factor (VEGF) and tunica interna endothelial cell kinase (TIE2), and stimulates PDAC angiogenesis (19, 20). On the other hand, TAMs will also secrete chemokines including CCL20 and CCL18, induce the upregulation of matrix metalloproteinase 9 (MMP9) and vascular cell adhesion molecule 1 (VCAM-1) expression in pancreatic cancer cells, and enhance metastasis (21, 22). Nevertheless, TAMs can activate cancer-associated fibroblasts to promote fibrosis and increase the difficulty of drug delivery (23). Xian et al. also reported that TAMs directly induced resistance to gemcitabine in PDAC cells (24). Furthermore, D’Erico et al. suggested that TAMs can induce regulatory T cell infiltration, thereby inhibiting the efficacy of chemotherapy (25). Therefore, TAMs are one of the most important regulators in the tumor microenvironment, including tumorigenesis, immune escape, interstitial fibrosis, angiogenesis, and chemoresistance.

## 3 Dendritic cells

Dendritic cells (DCs) were previously classified as belonging to the mononuclear phagocyte system, until Steinman et al. distinguished them from macrophages (26). DCs are generally divided into three categories: classical (cDC1 and cDC2), plasmacytoid cell (pDCs), and monocyte or inflammatory DCs. Functionally, cDCs are more prone to present antigens, pDCs are key in type I interferon (IFN) secretion during viral infection, and the monocyte-derived or inflammatory DCs are common in areas of inflammation (27).

DCs demonstrate considerable functional heterogeneity, even in the same cell population. Villani et al. divided DCs into six groups based on single-cell RNA-seq data (28). Nevertheless, they also pointed out that single-cell sequencing alone was insufficient to support the new DC definition and classification, and it was difficult to distinguish whether DCs were a class of cells in different functional states under the same cell group or a *bona fide* new cell population based on differences in gene expression, which requires verification *via in vivo* and *in vitro* experiments.

**Figure 3:**
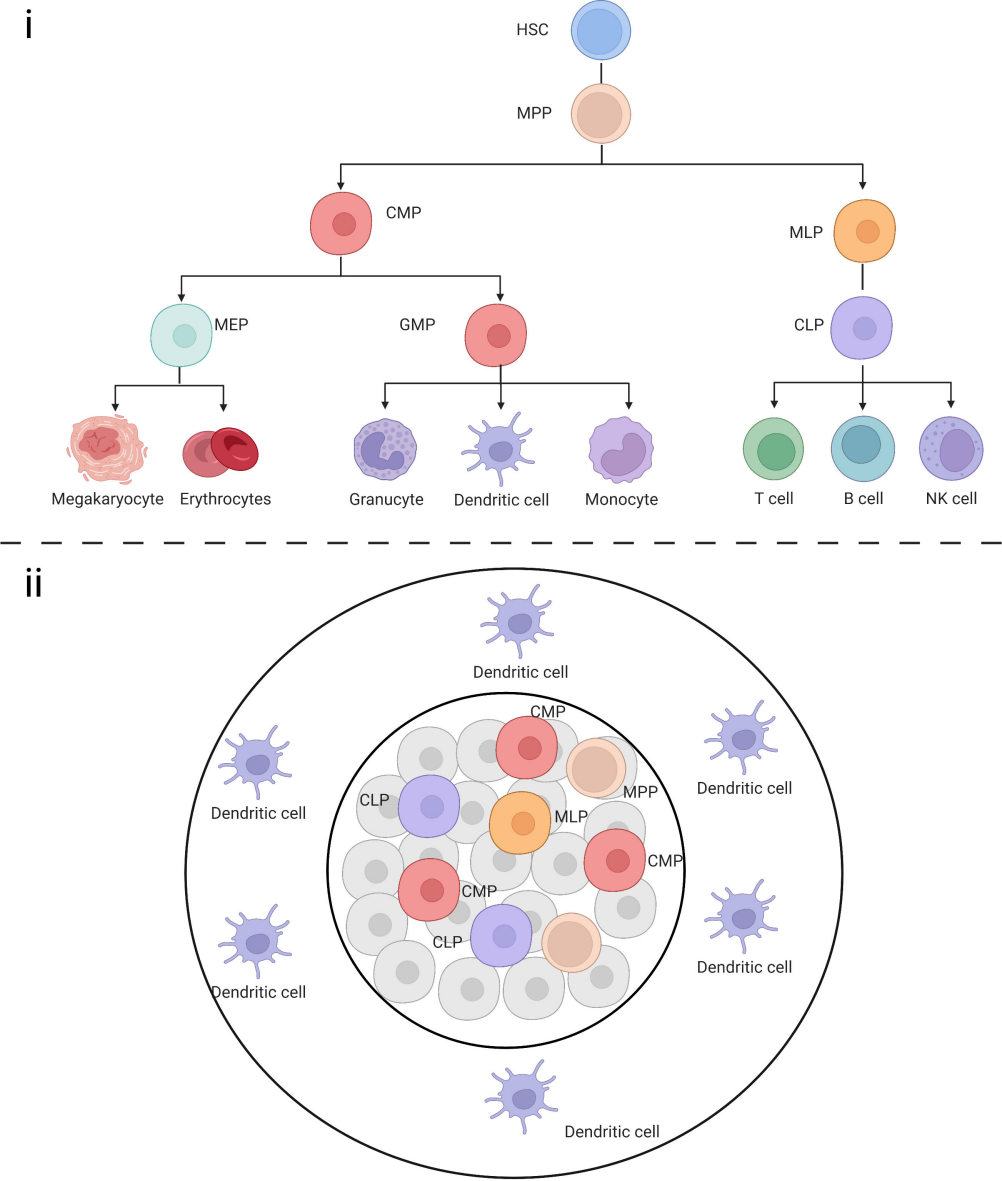
(i) Schematic representation of classical HSCs in a tree-like model. (ii) Multiple progenitor cells maintain the ability to differentiate into DCs.

It is believed that the cDCs are derived from common DC progenitors (CDP) and monocyte or inflammatory DCs originate from monocyte–macrophage DC progenitors, while the origin of pDCs remain a matter of debate. Previously, pDCs were thought to be of the myeloid lineage, whereas recent single-cell studies suggested that they mainly originate from the lymphoid lineage. It was believed that cell differentiation and development is a step-wise directed differentiation process, i.e., from HSC to common myeloid progenitors (CMP), granulocyte-macrophage progenitors (GMP), and monocyte-dendritic progenitor (MDP) to CDP, followed by migration to the peripheral organs to differentiate and mature into cDCs. However, recent research has challenged this view. Helft et al. reported that precursor cells with lymphoid commitment such as multi-lymphoid progenitors can also differentiate into DCs (29). Others have also suggested that the differentiation tendency of precursor cells with the same surface marker definition vary considerably. Therefore, unlike the previous directional development model of this tree-like structure (**Figure 3**), the development process in the human body might present multidirectional differentiation, including cells such as monocyte-dendritic progenitors and common DC progenitors, which maintain the directional differentiation of DCs while being able to differentiate into other cells when responding to external stimuli.

Compared with lung cancer, PDAC contains significantly fewer DCs (especially cDC1), which are mainly located on the tumor periphery (30). The maturation and antigen presentation-ability of cDC1 are relatively weak. This deficiency in cDC1 abundance and maturation are unique to pancreatic tumors, occurring from the early stage of pancreatic intraepithelial neoplasia and declining systematically and progressively as the disease progresses, which is caused by IL-6-mediated apoptosis (31). In PDAC, cDC1 and pDC infiltration in the tumor and tumor stroma was associated with better prognosis (32). High DC infiltration polarizes CD4^+^ and CD8^+^ tumor-infiltrating lymphocytes, while relatively poor DC abundance and antigen-presenting function impair the T cell response, leading to tumor immune escape. Therefore, DCs are indispensable as a key link in activating adaptive immunity. In a genetically engineered PDAC tumor model constructed by Hegde et al., featuring the poor infiltration and activation of DCs in the tumor, the pathological adaptive immune responses initiated by the expression of the engineered model neoantigen even accelerated the occurrence of PDAC through pro-fibrotic inflammation. By mobilizing cDCs into pancreatic lesions and maintaining their activation and function, this pro-fibrotic inflammation was reversed and PDAC progression was controlled. Based on this premise, a variety of DC-based treatment methods have emerged, including DC vaccines (33) and CD40 agonists that promote DC maturity (34). CD40 agonists effectively promote cDC1 maturation, while the Flt3 ligand (Flt3L) helps to increase cDC1 abundance, and the synergistic effect of the two promotes superior T cell activation and enhances anti-tumor immunity (31). Some trials of DC vaccines have also shown clinical effectiveness. For example, in a Phase I clinical trial of patients with PDAC, 10 patients were injected with autologous tumor lysate-loaded autologous monocyte-derived DCs. During a median follow-up period of 25 months, 7 patients have not experienced tumor recurrence or progression and no vaccine-related serious adverse reactions occurred, indicating good feasibility, safety, and clinical efficacy (33). The combination of CD40 agonists and DC vaccines also demonstrated clinical effectiveness by combining the advantages of both (34).

## 4 Mast cells

In mice, mast cells are classified as connective tissue mast cells (CTMCs) and mucosal mast cells (MMC). Li et al. used *Runx1^cre/EYFP^* and *Csf1r^cre/EYFP^* reporter mice to confirm that CTMCs in adipose tissue and the pleural cavity were mainly derived from early EMPs, while CTMCs in other organs were derived mostly from late EMPs. Therefore, mouse CTMCs are considered tissue-resident while mouse MMC are derived mainly from the bone marrow (35).

**Figure 4:**
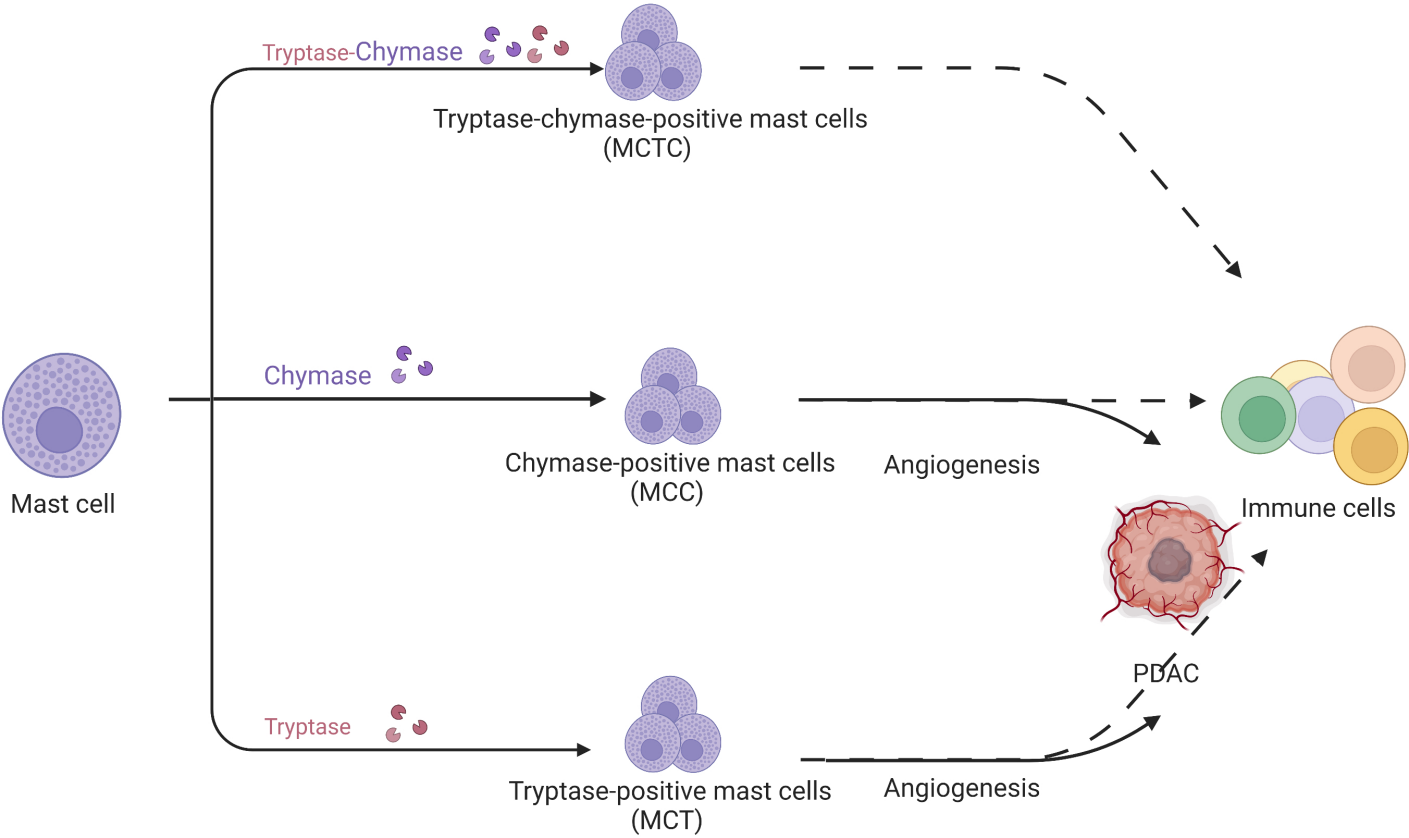
Mast cells are divided into tryptase/chymase-positive mast cells, MCCs, and MCTs according to their contents. MCCs and MCTs promote tumor development through their pro-angiogenic effects but their specific role in pancreatic cancer is unknown.

There are three mast cell populations in human: tryptase-positive mast cells (MCTs), chymase-positive mast cells (MCCs), and tryptase/chymase-positive mast cells (MCTCs) (**Figure 4**). Mast cells are important in infection (36), chronic inflammation (37), and metabolic diseases (38).

In human PDAC, Strouch et al. (39) firstly determined that MCTs were significantly increased in tumors and associated with poor prognosis. Guo (40) et al. and Ammendola et al. (41) demonstrated that MCTs were strongly associated with angiogenesis in PDAC. Ammendola et al. also confirmed that MCCs were positively associated with angiogenesis in pancreatic cancer (42). However, the above studies were based on the immunochemistry of PDAC specimens. Given the limitations of the method, immunochemistry cannot accurately assess the mast cell fraction and function in the PDAC microenvironment. Furthermore, most studies focused on tryptase-induced angiogenesis without the connection of mast cells and other immune cells in the tumor microenvironment. Some researchers believed that mast cell function cannot be reflected accurately by dividing mast cells according to their proteases. Therefore, referring to macrophage categorization, mast cells are divided into anti-tumor MC1 and pro-tumor MC2 based on TNF, granzyme B (GZMB), IL9, VEGF-A, and C-X-C motif chemokine ligand 8 (CXCL8) expression. Consequently, mast cell function remains ambiguous in PDAC.

Similar to macrophages, mast cells undergo prenatal development in two distinct waves: the first is from the yolk sac and fetal liver and the second is from the aorta-gonad-mesonephros region (43). Salomonsson et al. reported a group of Lin^−^CD34^hi^CD117^int/hi^FcϵRI^+^ cells in the peripheral blood in humans (44) that could further differentiate into mast cells in *in vitro* culture and were therefore considered mast cell precursors. However, considering the high heterogeneity of mast cells in peripheral tissue, some researchers proposed a different lineage model. Similar to DCs, a pool of non-committed stem cells might give rise to different types of mast cells (43).

With the further development of new technical methods, including single-cell sequencing and spatial transcriptomes, the identification and functional conversion of mast cells at different times and locations in pancreatic cancer occurrence and development will be further explored.

## 5 Myeloid-derived suppressor cells

Myeloid-derived suppressor cells (MDSCs) comprise a heterogeneous population of immature bone marrow cells that originate from bone marrow HSCs, differentiate into CMP and immature myeloid cells (IMP), and then migrate to peripheral organs and develop into GMP, which in turn form granulocytes, macrophages, or DCs (45). However, under pathological conditions, especially in tumors, a large number of cytokines are produced, including granulocyte-macrophage colony-stimulating factor (GM-CSF), granulocyte colony-stimulating factor (G-CSF), and macrophage colony-stimulating factor (M-CSF), which promote the formation of MDSCs through mitogen-activated protein kinase (MAPK) or phosphatidylinositol-4,5-bisphosphate 3-kinase (PI3K) pathways (46–48).

Based on their phenotypic and cell surface markers, MDSCs can be classified into two main types: polymorphonuclear MDSCs (or granulocytic (G-MDSCs)) and mononuclear or monocytic MDSCs. Human G-MDSCs are labeled as HLA-DR^-^CD33^+^CD11b^+^CD15^+^CD14^-^, and mononuclear or monocytic MDSCs are labeled as HLA-DR^low^CD11b^+^CD14^+^CD15^-^ (49, 50). MDSCs share surface markers with normal myeloid cell subsets, such as CD14, CD15, and CD33, and exhibit intrinsic heterogeneity; therefore, it is imperative to combine phenotypic characterization with functional assays for their identification.

**Figure 5:**
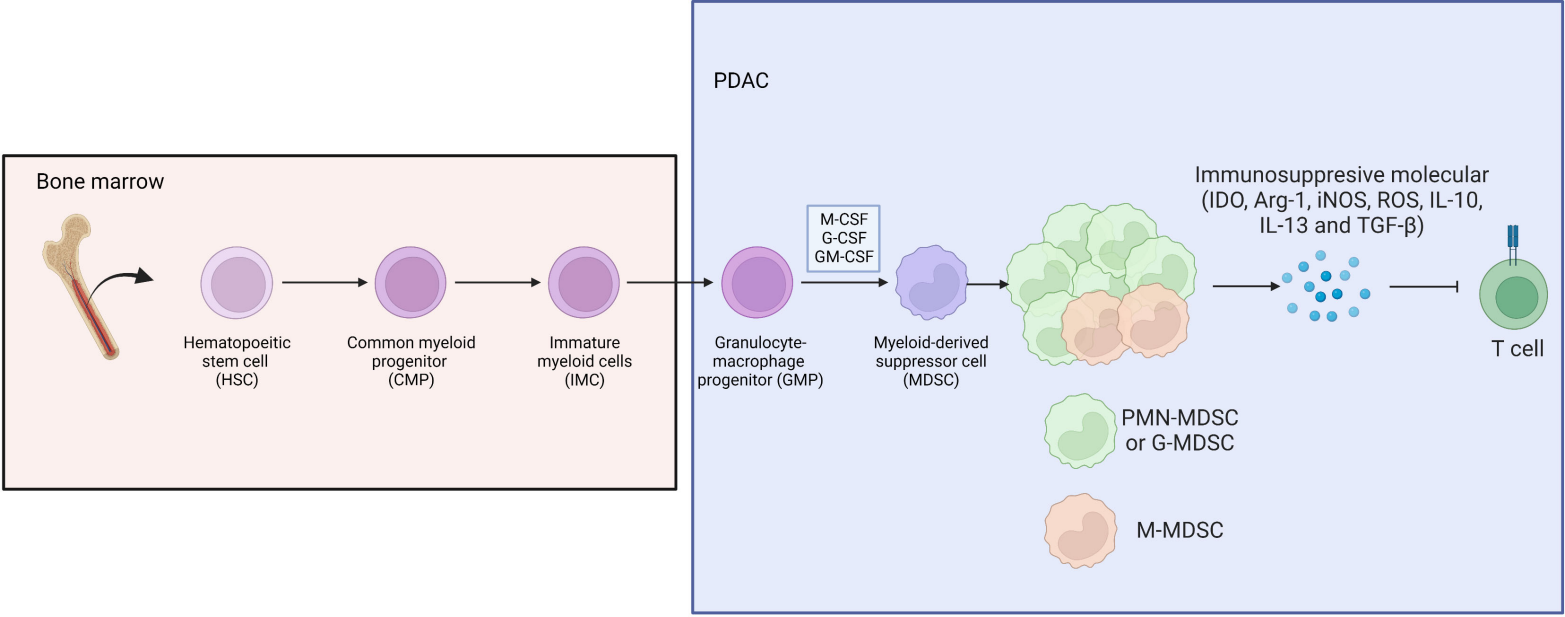
Bone marrow derived HSC differentiate to CMP, IMP and GMP, then develop into MDSC under stimulation of M-CSF, G-CSF and GM-CSF in PDAC, where G-MDSC occupied the majority of MDSC subpopulation.

MDSCs have a significant immunosuppressive function in the tumor immune microenvironment, which is mainly achieved by inhibiting the function of effector T lymphocytes, and this immunosuppressive effect is antigen-nonspecific. They can secret indoleamine 2,3-dioxygenase (IDO), arginase-I (Arg-1), inducible nitric oxide synthase (iNOS), reactive oxygen species (ROS), and several inhibitory cytokines (IL-10, IL-13 and TGF-β) (51) (**Figure 5**).

Khaled et al. phenotyped MDSCs in pancreatic cancer and found that G-MDSCs represented the majority of the MDSC subpopulation in circulation and in tumors, not mononuclear or monocytic MDSCs. And they found that Arg was significantly highly expressed in circulating G-MDSCs, which could dramatically inhibit the proliferation and activation of T cells (52). Enrichment of G-MDSCs further led to tumor progression, accompanied by a decrease in monocyte MDSC frequency. And it is worth noting that even after tumor resection, the MDSC cells in the peripheral blood of patients are still high, which may be related to tumor micrometastasis (53). Takeuchi et al. reported that the production of GM-CSF was significantly enhanced in various PDAC cell lines or PDAC tumor tissues of patients after chemotherapy, thereby inducing monocyte differentiation into MDSCs (54). The high heterogeneity of MDSCs means that selective depletion of MDSCs with antibodies for therapeutic effect remains elusive. However, Choueiry et al. reported that CD200 expression might expand MDSCs, and targeting CD200 could enhance the activity of checkpoint immunotherapy (55). In addition, Karakhanova et al. demonstrated that sildenafil could reduce the amount of chronic inflammatory factors (IL-1β, VEGF, and GM-CSF) and suppress immunosuppressive MDSC function, thereby restoring T cell antitumor reactivity and significantly prolonging survival in treated mice. Furthermore, Porembka et al. reported that zoledronic acid reduces MDSC prevalence and tumor growth in PDAC (56). However, the immunotherapy targeting MDSCs still needs more investigation.

## 6 Innate lymphoid cells

Innate lymphoid cells (ILCs) can be classified into natural killer (NK) cells, ILC1s, ILC2s, and ILC3s. As classic innate immune lymphocytes, NK cells have a different origin from ILC1s, ILC2s, and ILC3s, mainly originating from the bone marrow and constantly migrating through the circulation to the peripheral tissues (57, 58). The extreme fibrotic feature of PDAC means that the proportion of NK cell infiltration in PDAC tissue is very low (< 0.5%) and NK cell function in patients with pancreatic cancer is significantly inhibited compared with that in healthy subjects (59). NK cells express natural cytotoxicity receptors (NCRs: NCR1/NKp46, NCR2/NKP44, NCR3/NKP30), killer inhibitory receptors (KIRs), and other receptors, such as NK cell receptor D (NKG2D) and DNAX accessory molecule-1 (DNAM-1). In PDAC, NK cells exhibit impaired cytotoxicity receptors and increased IL-10 expression (60). As a novel immune receptor-targeted drug in pancreatic cancer, NKG2D is receiving increasing attention (61).

The mouse pancreas rarely contains ILC1s and ILC3s, wherein ILC2s are prevalent. ILC2s exhibit pronounced heterogeneity and high plasticity. Xu et al. reported that a higher percentage of ILC2s predicted poor prognosis in patients with hepatocellular carcinoma (62). Heinrich et al. reported that ILC2s were associated with better prognosis of patients with hepatocellular carcinoma (63). The discrepancy between these two studies might be because Xu et al. focused on KLRG1^-^ ILC2s, while Heinrich et al. concentrated on IL33 (alarmin)-activated KLRG1^+^ ILC2s.

**Figure 6:**
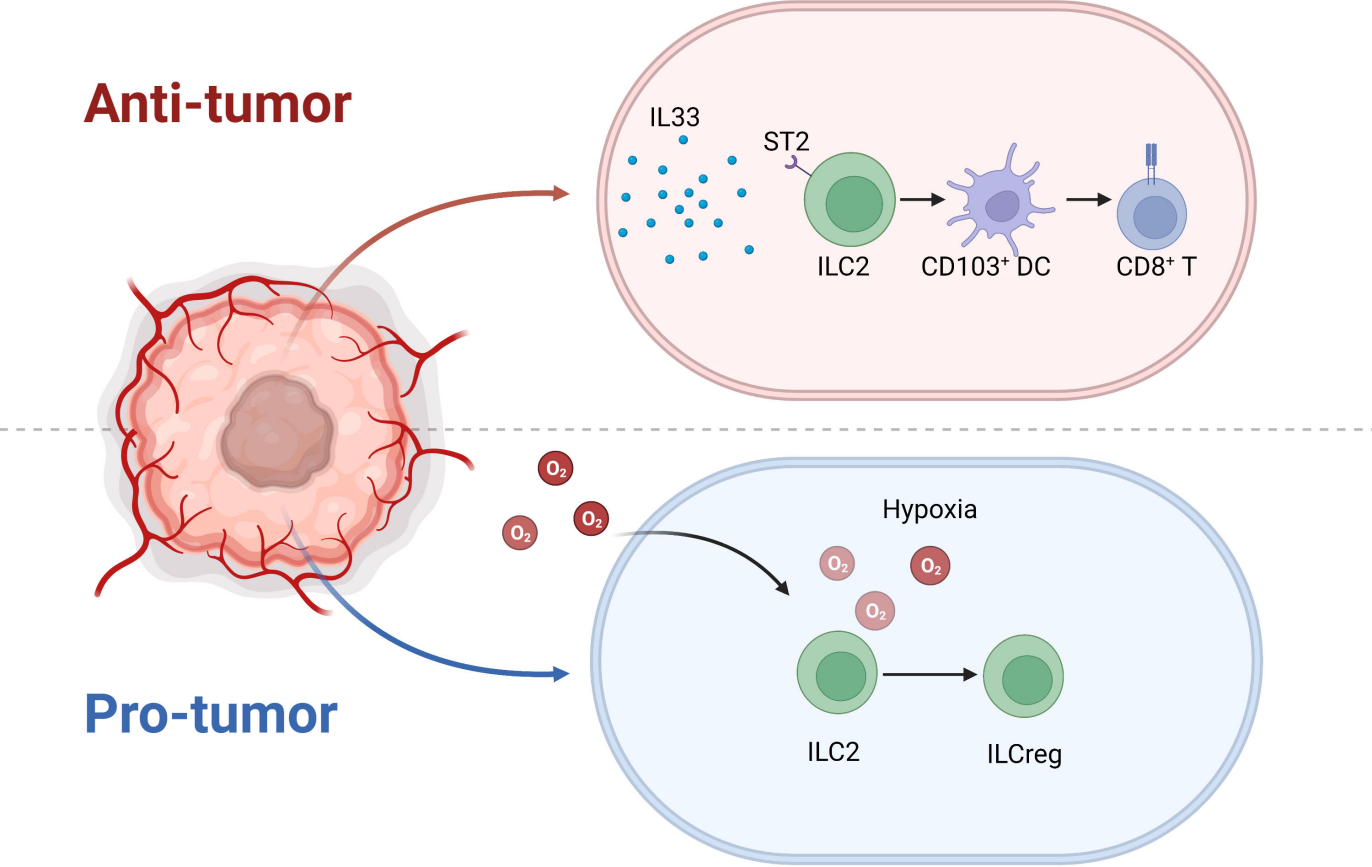
Under IL33 stimulation, ILC2 recruits CD103^+^ DCs and prime CD8^+^ T cells to inhibit tumor progression. Nonetheless, the extreme hypoxic environment of pancreatic cancer induces ILC2 conversion to ILCreg.

Moral et al. demonstrated that IL33-activated ILC2s recruited CD103^+^ DCs to activate CD8^+^ T cells in PDAC, which predicted better survival (64). However, Alam et al. reported that the mycobiome upregulated IL33 then activated ILC2s in PDAC, which was associated with poor prognosis (65). Therefore, the function of ILC2 in pancreas is still on the debate. Our research confirmed that IL33-activated ILC2s shrank tumors in a mouse model. However, the tumor microenvironment is complicated, such that not only are molecules such as IL33 present, but so are extreme physical conditions, such as hypoxia. Furthermore, we detected no significant association between IL33 and overall survival or disease-free survival in the Cancer Genome Atlas PDAC data (1). Therefore, IL33 activation in ILC2 function in cancer might be overestimated. IL33-activated ILC2s have a well-established role in inflammation, especially in asthma (66). However, in the tumor microenvironment, the classical suppression of tumorigenicity 2 (ST2)–IL33 pathway might not be the dominant pathway. Our RNA-seq data demonstrated that hypoxia induced regulatory ILCs from an ILC2 population in PDAC, which differed from the *Id3*-expressing regulatory ILCs in the mouse intestines (67) and the retinoic acid-induced regulatory ILCs in patients with asthma (68) (**Figure 6**). Therefore, thorough investigation of the PDAC tumor microenvironment complexity to clearly demonstrate how ILC2s transition when encountering different signals is still required.

Similar to tissue-resident macrophages, the maintenance and expansion of most ILCs in peripheral tissues is independent of the bone marrow. ILC precursor cells migrate to the peripheral tissues during the embryonic and postnatal developmental window and their proportions differ greatly in different peripheral organs and tissues (69). Huang et al. used the CD45.1 and CD45.2 mouse parabiosis model to prove that there was a considerable proportion of immune cells in the circulation from each mouse type; although ILCs were hardly exchanged with each other in the peripheral organs (70). However, because of the abundant proliferation of ILCs in infection, it is speculated that a group of ILC precursor cells is present in the peripheral organs. Zeis et al. reported on a population of IL18R1^+^ST2^-^ ILC2 precursor cells in the lung that could differentiate into ILC2s *in vitro* and *in vivo* (71).

However, because of its relatively “clean” property, the non-cancerous pancreas contains almost no lymphocytes. Therefore, it is difficult to detect the possible ILC2 progenitor cells in the pancreas. In PDAC, 20–30% of CD45^+^ cells are CD103^+^. Therefore, circulatory ILC2s might exist in patients with PDAC (1). Lim et al. reported the presence of CD117^+^ multi-potent ILC progenitors in human peripheral blood, which gave rise to all ILC subsets (72). Moreover, Huang et al. reported the existence of inflammatory ILC2s in mice that could migrate from the intestinal lamina propria to other tissues under the induction of IL25 or helminth-induced inflammation and replenish natural ILC2s in various organs (70). Therefore, the function and proportion of tissue-resident and circulating ILC2s in PDAC still requires further investigation.

## 7 Conclusion

Pancreatic cancer is one of the most malignant tumors and its incidence increases year-by-year as lifestyles change. Nevertheless, the 5-year survival rate remains at approximately 10% and there has been no significant improvement compared with 10 years ago (73, 74). Therefore, there is an extremely urgent need to find new treatments. Unfortunately, unlike other digestive system tumors, immune checkpoint antibodies are not successful to treat pancreatic cancer. Nonetheless, innate immunity might be a promising target.

Accumulating research evidence demonstrates that most innate immune cells in the peripheral organs are more inclined to be tissue-resident rather than classically bone marrow-derived. The unsatisfactory efficacy of current immunotherapy regiments in PDAC prompted us to investigate the roles of innate immune cells in PDAC. A reasonable therapeutic schedule should focus on not only enhancing the function of adaptive immunity cells, but also on activating innate immunity in the tumor microenvironment. The application of DC vaccines or their combination with CD40 agonists have achieved promising results in early phase clinical studies. However, targeting the innate immune system still faces huge challenges and requires more detailed exploration to bring hope for a breakthrough in pancreatic cancer immunotherapy.

## Author contributions

LY and SS contributed to the conception of the study and wrote the manuscript. WC reviewed the manuscript and provided suggestions for revision. All authors contributed to the article and approved the submitted version.
